# Proteomics analysis of serum small extracellular vesicles for the longitudinal study of a glioblastoma multiforme mouse model

**DOI:** 10.1038/s41598-020-77535-8

**Published:** 2020-11-24

**Authors:** Federica Anastasi, Francesco Greco, Marialaura Dilillo, Eleonora Vannini, Valentina Cappello, Laura Baroncelli, Mario Costa, Mauro Gemmi, Matteo Caleo, Liam A. McDonnell

**Affiliations:** 1 NEST Laboratories, Scuola Normale Superiore, 56127 Pisa, Italy; 2 Fondazione Pisana per la Scienza ONLUS, 56107 San Giuliano Terme, PI Italy; 3 Institute of Life Sciences, Sant’Anna School of Advanced Studies, 56127 Pisa, Italy; 4 CNR, Neuroscience Institute, 56124 Pisa, Italy; 5 Fondazione Umberto Veronesi, 20122 Milano, Italy; 6 Istituto Italiano di Tecnologia, Center for Nanotechnology Innovation @NEST, 56127 Pisa, Italy; 7 IRCCS Fondazione Stella Maris, 56018 Calambrone, PI Italy; 8 Department of Biomedical Sciences, University of Padua, 335122 Padua, Italy

**Keywords:** Proteomics, Tumour biomarkers, Mass spectrometry, Proteomic analysis, Proteomics

## Abstract

Longitudinal analysis of disease models enables the molecular changes due to disease progression or therapeutic intervention to be better resolved. Approximately 75 µl of serum can be drawn from a mouse every 14 days. To date no methods have been reported that are able to analyze the proteome of small extracellular vesicles (sEV’s) from such low serum volumes. Here we report a method for the proteomics analysis of sEV's from 50 µl of serum. Two sEV isolation procedures were first compared; precipitation based purification (PPT) and size exclusion chromatography (SEC). The methodological comparison confirmed that SEC led to purer sEV’s both in terms of size and identified proteins. The procedure was then scaled down and the proteolytic digestion further optimized. The method was then applied to a longitudinal study of serum-sEV proteome changes in a glioblastoma multiforme (GBM) mouse model. Serum was collected at multiple time points, sEV’s isolated and their proteins analyzed. The protocol enabled 274 protein groups to be identified and quantified. The longitudinal analysis revealed 25 deregulated proteins in GBM serum sEV's including proteins previously shown to be associated with GBM progression and metastasis (Myh9, Tln-1, Angpt1, Thbs1).

## Introduction

Intercellular communication via the secretion of extracellular vesicles (EVs) is now established as a major pathway in disease progression^1,2^. EVs are a heterogeneous population of vesicles naturally released from cells^3^. EVs are secreted by most cells types, including cancer cells and are present in nearly all body fluids^4,5^. EVs released from cancer cells contain tumour derived material, and thus many studies have focused on their role in the communication between cancer cells, stroma and the immune system, as well as a potential source of biomarkers^6,7^.

It is now well established that small extracellular vesicles (sEV’s, diameter < 150 nm) play an important role in transport and communication processes^8^. Small extracellular vesicles offer significant advantages over circulating tumour cells (CTCs) as biomarkers, including their greater abundance and stability^9^. Moreover sEV's have been identified from tumours that did not release detectable CTCs^10^. sEV's represent an opportunity for minimally invasive biomarker research, for instance for cancer diagnosis and monitoring^11^. Glioblastoma multiforme (GBM) is the most frequent and malignant primary tumour of the brain, and has one of the lowest 5-year survival rates of all human cancers^12–14^. One of the reasons for the poor outcome is the late diagnosis, which is based on neuroimaging techniques and tissue biopsies once symptoms are already manifest^15^. sEV’s play an important role in the brain tumor environment, regulating the transfer of oncogenic proteins, receptors and soluble molecules that support the tumor progression^16^. Glioblastoma sEV’s release angiogenic factors and promote cell proliferation through activation of the PI3K/AKT pathway^17,18^. The prominent role of sEV’s in GBM as well as their ability to cross the blood brain barrier makes them good candidates for the identification of circulating biomarkers to enable earlier diagnosis and treatment^19^.

Xenograft tumour mouse models are widely used for the study of disease progression, biomarker discovery and drug testing, on account of their shorter lifespan and rapid disease progression^20,21^. The increasing requirement to replace, reduce and refine (3Rs) animal research places great emphasis on ensuring maximum information content from each experiment^22^. Longitudinal measurements of individual animals reduce the impact of inter-individual variability by examining the molecular changes for each individual, rather than comparing populations of animals for each time point. The longitudinal analysis of sEV's from individual mice would improve data quality through reducing inter individual variability, as well as better satisfy the 3Rs animal welfare requirement. The longitudinal analysis of circulating sEV’s in rodent models is challenging owing to the limited serum/plasma that may be obtained at each time point; for instance just 75 µl of serum may be obtained every 14 days for adult mice^23^.

The analysis of the proteome of serum/plasma sEV's requires methods that are able to isolate sEV's from the highly complex chemical background of serum while maintaining sEV integrity. This includes the separation of sEV's from protein aggregates, lipoproteins, and the abundant proteins present in serum/plasma^24,25^. The current gold-standard for sEV isolation is based on differential ultracentrifugation^26^, however the sample volume needed is typically several millilitres^27,28^. Precipitation and SEC based methods have been reported for sEV proteomics studies that use relatively low volume samples^29–31^. Munson et al. used SEC and LC–MS/MS based proteomics to investigate the exosome proteome following asbestos exposure^30^ using only 200 µl of serum. Here we report the development of a workflow for the proteomics analysis of sEV's from just 50 µl of mouse serum and demonstrate its ability to perform longitudinal analysis of GBM development in individual mice.

## Results and discussion

**Figure 1:**
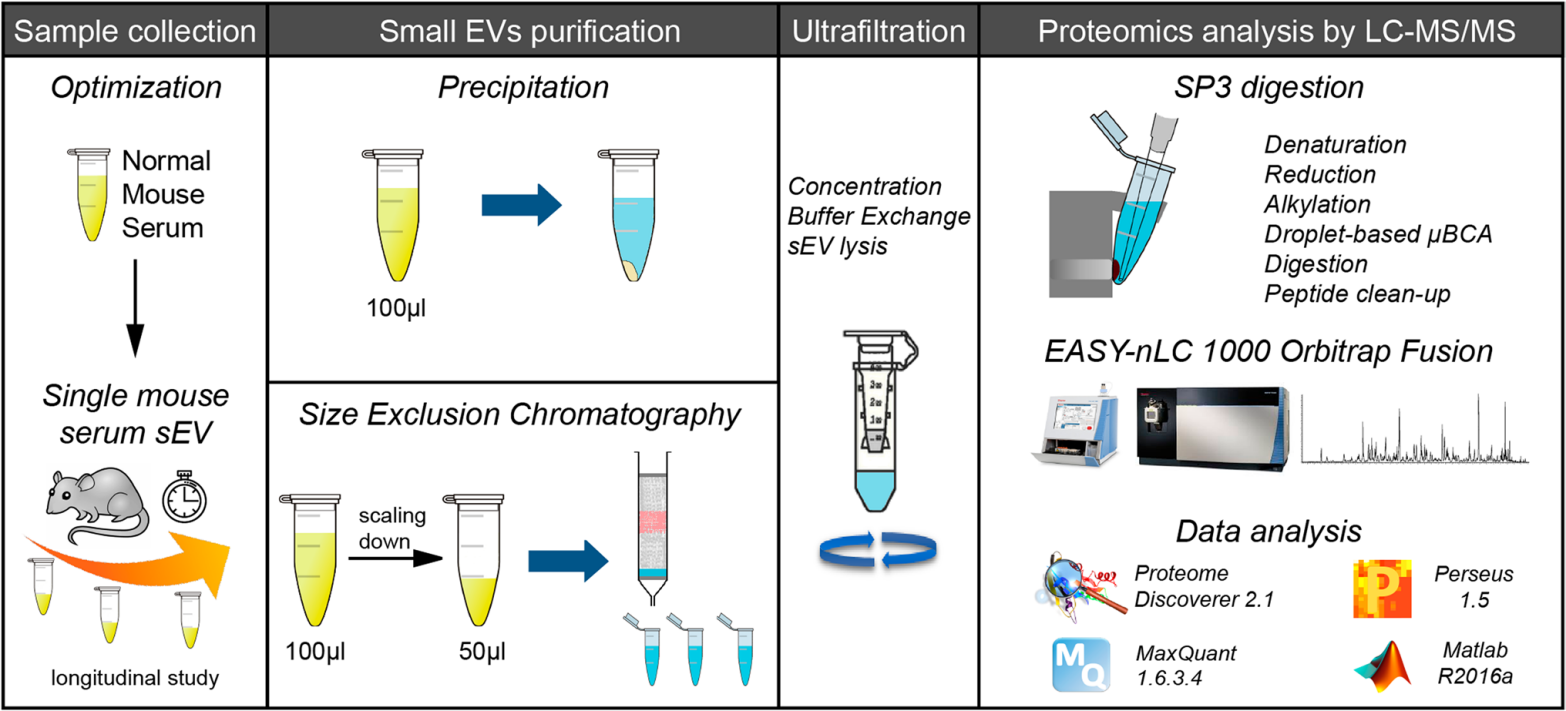
Experimental workflow. Comparison between two sEV isolation procedures: precipitation (PPT) and Size Exclusion Chromatography (SEC) using 100 µl of mouse serum. SEC was then scaled down on vesicles isolated from 50 µl of serum. As a proof of concept, the ultrasensitive microproteomics workflow was then applied to a longitudinal study of a glioblastoma multiforme mouse model. Purified sEV’s were concentrated and lysed on protein concentrator spin filters; then extracted, quantified and digested with a modified SP3 protocol. Peptides were analyzed by nLC-MS/MS, and the data analyzed with Proteome Discoverer 2.1 and MaxQuant software. Statistical analysis was performed using Perseus and Matlab.

A workflow was developed for the isolation and proteomics analysis of sEV’s from low volumes of serum for the longitudinal analysis of GBM development in individual mice. The study design is illustrated in Fig. 1 and described in detail in the “Materials and methods” and Supplementary Information sections.

The first objective was to compare two sEV isolation methods that have been reported for serum volumes in the range 200–500 µl (still appreciably greater than the 50 µl needed for the longitudinal analysis of individual mice), namely SEC and precipitation (PPT). Both procedures were performed in triplicate using 100 µl serum aliquots.

**Figure 2:**
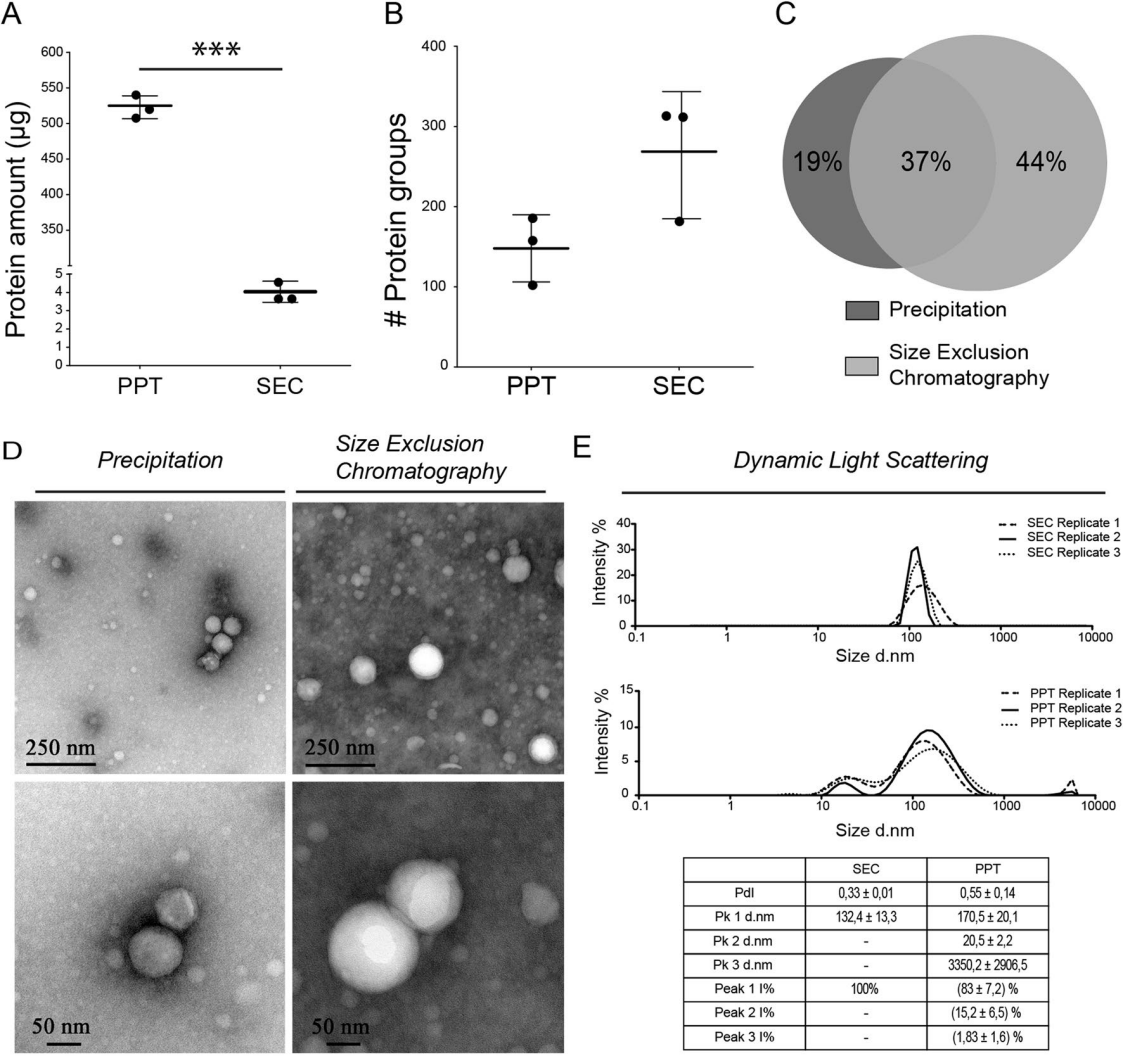
Comparison of PPT and SEC sEV isolation methods. (**A**) Extracted protein amount from SEC- and PPT-sEV’s. The number of identified protein groups is described in B. Welch’s test: *p < 0.05, **p < 0.01, ***p < 0.001. Data are mean ± SD. (**B**) Number of identified protein groups of SEC- and PPT-sEV’s. Data are mean ± SD. (**C**) Venn diagram showing overlap of proteins identified from the SEC and PPT preparations. Dark grey circles indicate PPT IDs, light grey circles indicate SEC IDs. (**D**) Transmission electron microscopy images at low and high magnification of negative staining sEV’s obtained by PPT and by SEC. (**E**) Dynamic light scattering analysis and relative dimension distribution obtained for PPT-EV and SEC-EV. Data are mean (nm) ± SD.

The total protein amount that could be extracted from the sEV’s isolated by PPT was greater than that isolated by SEC, 523 ± 16 µg and 4 ± 0.58 µg respectively, Fig. 2A. LC–MS/MS proteomics analysis revealed that the SEC-EV method led to the identification of more proteins than PPT-EV, 334 ± 28 versus 274 ± 21 protein groups respectively (Fig. 2B, Supplementary Dataset SD1). Just 37% of the total number of identified proteins were common to both datasets, 19% were exclusive to PPT-EV and 44% exclusive to SEC-EV, Fig. 2C. A comparison with the proteins that are expected to be present in sEV's (MISEV 2018 guidelines^3^) demonstrated that the SEC-EV proteins included a larger number of vesicle markers, which included both membrane proteins (tetraspanins and integrins) and cytosolic proteins (Sdcbp, Hspa8). A comparison with the EV databases ExoCarta Top 100^32^ and EVpedia^33^ also confirmed the higher purity of the SEC-EVs. The results of these comparisons are summarized in Supplementary Table S2, and the list of ExoCarta Top 100 proteins identified here are reported in Supplementary Table S3. It should be noted that the results reported in Fig. 2 and Supplementary Tables S2 and S3 are conservative, as they were calculated using only those proteins identified in all technical replicates; many other EV proteins were identified in the SEC-EV samples but not in all replicates (e.g., Cd9, Itga2, Anxa4, Anxa5, Anxa7, Vamp8). Finally, a gene ontology analysis of the identified proteins also demonstrated the higher purity of the sEV's isolated by SEC, whereas 'extracellular exosomes' (FDR: 5,8E-110) was identified as the first cellular component category for the SEC-EV proteins, it was the fourth category for the PPT-EV samples and was significantly below the 'extracellular region', 'blood microparticle', and 'extracellular space' categories, Supplementary Table S4. Dynamic Light Scattering (DLS) and Transmission Electron Microscopy (TEM) analyses were also performed to characterize the sEV preparations, and which further supported the LC–MS/MS result that SEC provided purer sEV's. The TEM analysis (Fig. 2D) revealed that both SEC and PPT isolated intact vesicles, the PPT-EVs also included large features that could not be resolved by TEM and that were not present in the SEC-EV samples. The DLS measurements (Fig. 2E) revealed that the SEC-EVs were characterized by a single peak, centred at 130 nm hydrodynamic radius, whereas the PTT-EVs had three distinct populations, centred at 20 nm, 170 nm and 3350 nm hydrodynamic radius.

### Optimization of proteolysis of SEC-EVs isolated from 50 µl of serum

**Figure 3:**
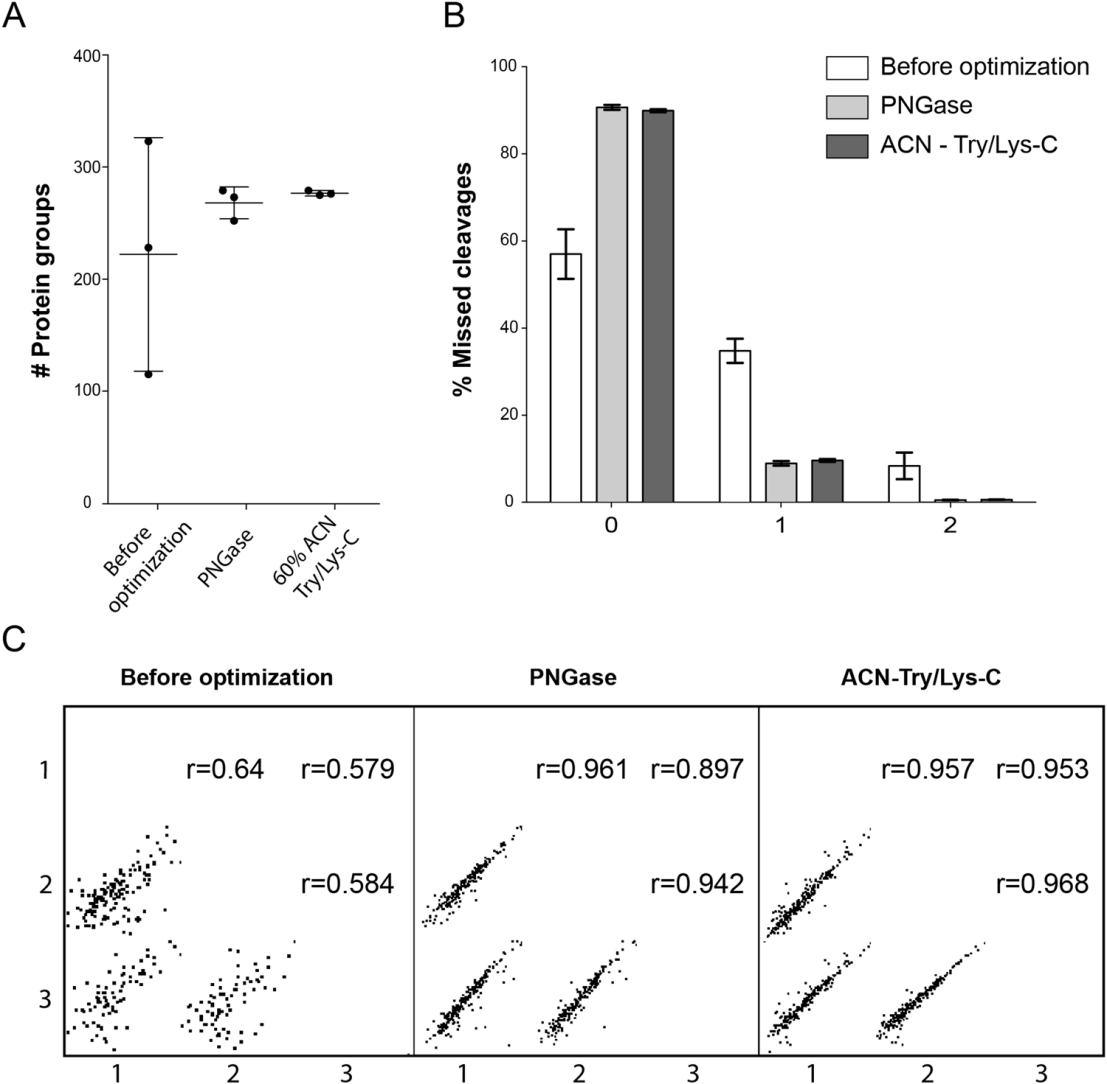
Optimization of proteolytic digestion of sEV proteins obtained from 50 µl of serum. (**A**) Number of identified protein groups obtained using an 18 h incubation with a Try/Lys-C mixture, and using two supplemental digestion strategies in which the initial 18 h incubation was changed to a 16 h incubation followed by supplemental addition (2 h. incubation) of the *N*-glycosidase PNGase F or additional Try/Lys-C in 60% acetonitrile. (**B**) Percentage of peptide spectral matches (PSMs) that are zero-missed-cleavage peptides, which are considered an indication of digestion performance. Data are mean ± SD. (**C**) Scatter plots between triplicates of different digestion conditions (before optimization, PNGase, and ACN) and relative Pearson correlation coefficients.

The comparison of the SEC-EV and PPT-EV isolation methods by LC–MS/MS, DLS, and TEM indicated that the SEC-based system provided sEV samples with less serum protein background and less aggregation. The SEC based isolation method and proteomics analysis were then further optimized to enable serum sEV proteomics analysis from 50 µl mouse serum. Initial tests resulted in the extraction of 2.6 ± 0.26 µg of protein, which led to the identification of 222 ± 104 protein groups, Fig. 3A. Close examination of the data indicated incomplete proteolysis, as the percentage of zero missed-cleavage peptides (57%) was significantly lower than the 90% threshold we routinely use to quality control the LC–MS/MS data (Fig. 3B).

The degree of proteolytic digestion was increased by the supplemental addition of more enzyme (see Supplementary Table S5 for experimental conditions). The supplemental addition of more trypsin/lys-c increased the number of identified proteins and reduced variability (Fig. 3C), with 277 ± 2 protein groups identified from the sEV’s isolated from 50 µl mouse serum. Similar results were obtained following the supplemental addition of the glycosidase PNGase F (Supplementary Dataset SD1). The supplemental addition of enzyme increased the number of identified proteins, increased the degree of proteolysis, and increased technical reproducibility. We then applied the SEC-EV workflow on 50 µl serum and 50 µl plasma from the same mice (n = 3). The serum-EV derived proteins exhibited greater reproducibility than the plasma-EV derived proteins; a principal component analysis (PCA) revealed that the main difference was due to the presence of fibrinogen proteins in the plasma-EV samples. The results of this experiment are provided in the Supplementary Information (Supplementary Results SR1, Supplementary Fig. S2).

### Longitudinal analysis of serum sEV’s from a mouse model of glioblastoma multiforme

The proteomics workflow for the characterization of the sEV proteome from low serum volumes enabled its application to a longitudinal study of GBM progression in an induced GBM model. The sEV proteins from GBM inoculated mice (n = 4) at multiple time points were compared using only 50 µl of serum from each mouse at each time point. Serum was also collected from three control mice (see “Materials and methods” for details). The SEC-EV procedure enabled the extraction of 1.7 ± 0.5 µg of sEV proteins. Following digestion using supplemental addition of Trypsin/Lys-C and LC–MS/MS analysis 274 protein groups were identified and quantified, and which included proteins commonly identified in sEV’s: integrins (α6, α-IIb, β1, β3), Anxa2, Gapdh, Adam10, Hspa8, and Tgfb1 (Supplementary Dataset SD2).

**Figure 4:**
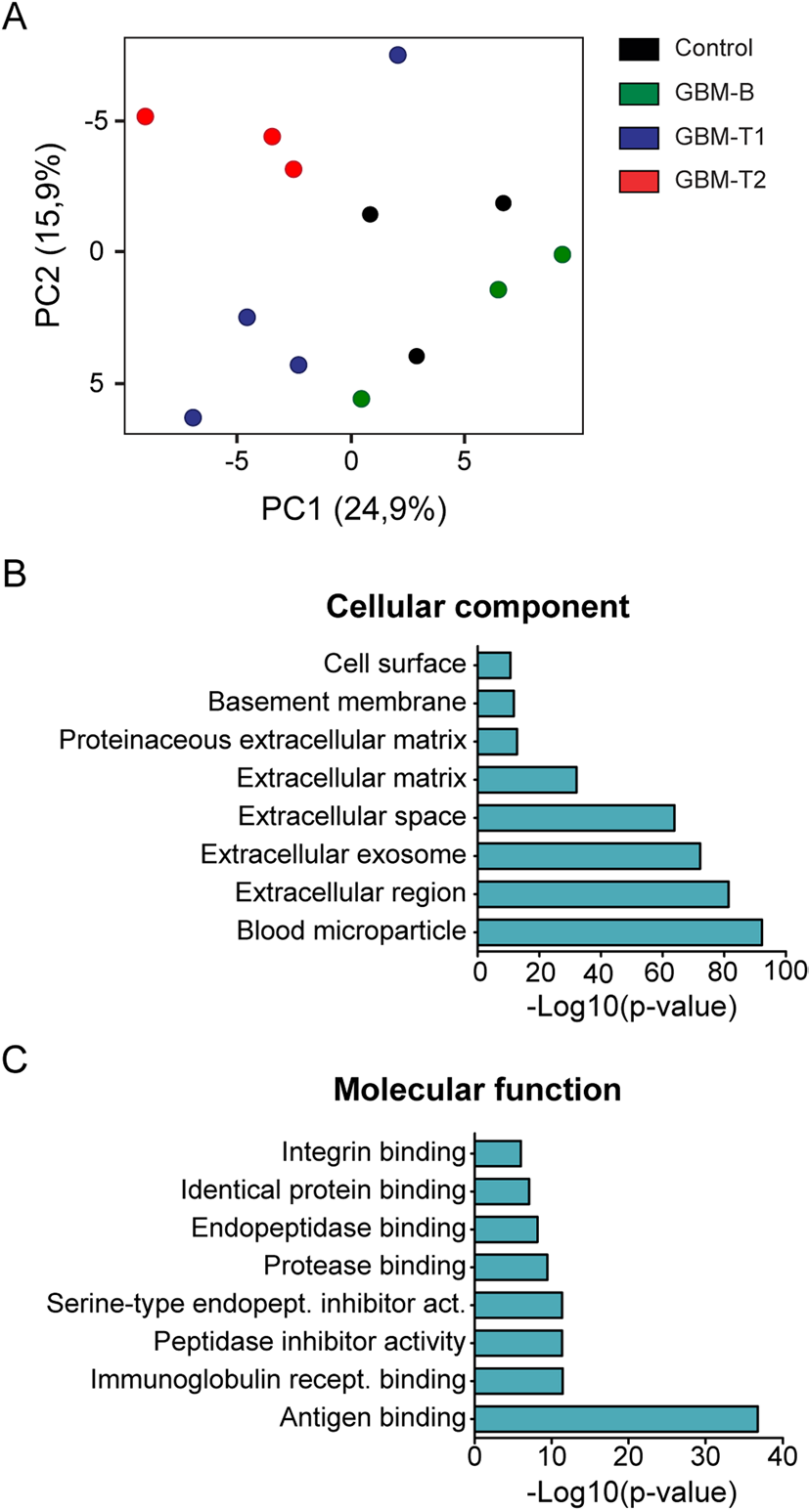
Longitudinal analysis of serum sEV’s from a mouse model of glioblastoma multiforme. (**A**) PCA score plot co-localizes the GBM-baseline time-point with the control animals (green and black datapoints respectively), and distinguishes between these and the GBM-T1 and GBM-T2 timepoints (blue and red, respectively). (**B**) Cellular component GO enrichment analysis of the sEV proteins identified using the DAVID database^62^. (**C**) Molecular function GO enrichment analysis of the sEV proteins identified using the DAVID database.

PCA of the quantitative proteomics data separated the GBM-mouse-sEV proteins (GBM-T1 and GBM-T2) from the healthy animals (GBM-baseline and controls), Fig. 4A, indicating that the principal difference in sEV protein cargo was related to GBM. A gene ontology (GO) enrichment analysis of the identified protein groups was performed using the total *mus musculus* genome as background. The analysis revealed the following GO cellular components were all enriched (Fig. 4B): blood microparticles (count: 68, FDR: 5,4E-93); extracellular exosomes (count: 153, FDR: 6,3 E-73); and cell surface (count: 36, FDR: 2,1E-11). Molecular functions related to protein binding were also strongly enriched (Fig. 4C). Extracellular exosomes are defined as sEV’s that originate from late endosomes^34^; in this study the ‘extracellular exosome’ category was strongly enriched. We investigated sEV biogenesis by examining the list of identified proteins for those associated with the late-endosomal pathway. Proteins belonging to the endosomal sorting complexes required for transport (ESCRT), which are involved in exosome biogenesis, were not identified in this study. Here the high complexity of the serum sample and the very small serum volume available at each time point of the longitudinal investigation meant that lower abundant proteins were not identified. Nevertheless, there was evidence for the endosomal origin of the sEV’s, specifically the proteins Ifitm3, Lamp1, Egfr, Trf, ApoE, Anxa2, Hspa8 are associated with late endosomes (GO0005770) and Pigr, Itgb1, Tfrc, Cltc, Plin3, Lrp1, Hrg are associated with endosomes (GO0005768). Note: In this study the term sEV was used instead of exosome because, according to guidelines published by the International Society of Extracellular Vesicles, the term exosome should only be used if it can be explicitly demonstrated that they have a late endosomal origin^3,35^.

### Longitudinal sampling better resolves molecular changes associated with GBM development

**Figure 5:**
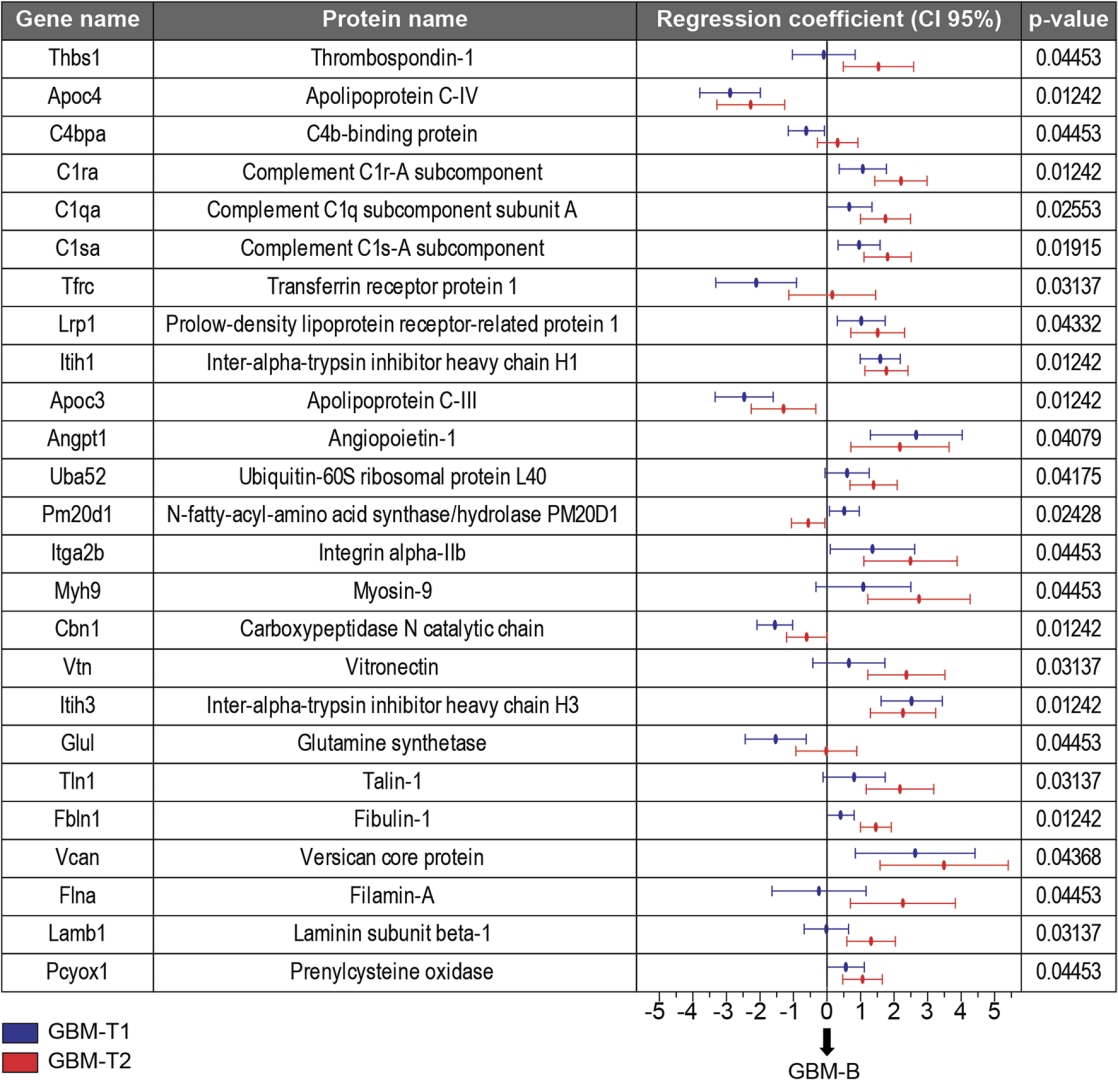
Linear mixed effects analysis of the longitudinal analysis datasets. List of the significantly deregulated proteins with relative p-values and regression coefficients. The regression coefficients (GBM-T1 in blue and GBM-T2 in red) are plotted with a confidence interval of 95%.

A comparison of the abundances of the sEV proteins from symptomatic GBM (T2) mice with those from independent control animals using a Student’ t-test resulted in 16 protein groups with a p-value < 0.05, but which were no longer significant after an FDR correction because of the small number of animals (Supplementary Dataset SD3). Such group-wise analyses compare mean protein intensities for each class, and large numbers of animals/patients are needed to counter the substantial biological variation. The longitudinal sampling of individuals enables the changes in protein expression to be differentiated from inter-subject variability. The longitudinal GBM data were analysed using a mixed-effects model (Supplementary Methods S5), in which the model was individually fitted to each protein. The model better accounts for inter-subject variability since it includes an effect for each time-point and a variable intercept for each mouse, i.e. the baseline level for each mouse is allowed to vary but the changes associated with disease progression should be consistent. The model led to the identification 25 significantly deregulated protein groups (Supplementary Dataset SD4). Proteomics data of the deregulated proteins such as accession number, sequence coverage, peptide score and MW are available in Supplementary Table S6. The proteins that exhibited a statistically significant time effects are reported in Fig. 5 with their relative p-value and regression coefficients for time points T1 and T2. Note that since the data were log2 transformed during data pre-processing (see Supplementary Methods S5) the regression coefficients can be considered as fold changes with respect to the baseline. 20 out of the 25 significantly deregulated proteins are annotated as glycoproteins (KW-0325); it is well known that sEV-proteome is highly glycosylated^36^, glycans play a major role in sEV cellular recognition and in the uptake of sEVs by recipient cells^37,38^. Among the 25 proteins, Lrp1, Cpn1, Mhy9, and Tln1 are associated to the plasma membrane (GO0098590). The low-density lipoprotein receptor-related protein 1 (Lrp1), positively regulates tau protein uptake and spread between neurons^39^, the tau protein is a known mediator of microtubule-dependent migration of glioblastoma cells^40^. Myosin IIA (Myh9) is involved in cell migration and is required for tumour invasion and metastasis^41,42^. Talin-1 (Tln1) has previously been reported in neuroblastoma derived exosomes^43^, is highly expressed in glioma tissues^44,45^ and promotes the motility of glioma cells^46^. Thbs1, Itga2b and Tfrc belong to the external side of plasma membrane (GO0009897). Thrombospondin (Thbs1) is involved in the tumour microenvironment and is known to be more highly expressed in glioblastoma^47,48^. Moreover, Thbs1 was found to be upregulated in hypoxic GBM-exosomes^49^. Transferrin receptor protein 1 (Tfrc, CD71) is known to be expressed on the surface of neuroblastoma exosomes^43^, and has been reported as a marker for radio-resistant GBM cells^50^. Angpt1, Vtn, Vcan and Lamb1 are known mediators of cell adhesion (GO0007155) and are all glycoproteins that likely contribute to sEV-interaction and cellular uptake. Moreover, Itga2b, Vtn, Lamb1, Thbs1 and Angpt1 are positive regulators of cell proliferation, angiogenesis, and cell survival through the extracellular activation of the PI3K/AKT pathway and were found to be significantly increased during GBM progression. Figure 5 shows that some of the deregulated proteins (Apoc4, C1ra, C1sa, Lrp1, Itih1, Apoc3, Angpt1, Itga2b, Itih3, Vcan) were significantly deregulated at both T1 and T2, meaning that protein changes were also detected at a presymptomatic stage; these proteins could be considered as candidate biomarkers for earlier detection but will first require further validation.

### Concluding remarks

These results demonstrate that the combination of SEC-EV isolation with SP3 bottom-up proteomics enabled the longitudinal analysis of sEV proteins in individual mouse models of GBM, and enabled the identification of proteins associated with tumor development and candidate biomarkers for the presymptomatic stage.

## Methods

### Materials

Normal Mouse Serum (10 mL), microBCA protein assay kit, Pierce Concentrator 3 K MWCO 0.5 mL, and Total Exosome Isolation from plasma were purchased from Thermo Fisher Scientific (Rockford, IL). Trypsin/Lys-C mix Mass Spec grade was purchased from Promega (Madison, WI) and PNGase F PRIME from N-Zyme Scientifics (Doylestown, PA). Size Exclusion Chromatography columns (qEV/70 nm single) were purchased from IZON (Christchurch, New Zealand) and 0.22 μm centrifugal filters were purchased from Merck (Darmstadt, Germany). All other reagents and solvents were purchased from Sigma-Aldrich (St. Louis, MO).

### Mice and tumour induction

Adult C57BL/6J mice were bred at the CNR Institute of Neuroscience animal facility and housed in a 12 h’ light/dark cycle, with food and water available ad libitum*.* All experimental procedures were performed in conformity to the European Communities Council Directive 86/609/EEC and were approved by the Italian Ministry of Health (DLSG 26/2014 Authorization No. 260/2016-PR). The murine glioma GL261 cell line was grown as specified in Vannini et al.^51^. Tumour cell inoculation was performed by stereotaxic guided injection; more details are available in the Supplementary Information (Supplementary Methods S1).

### Mouse serum and plasma collection

The retro-orbital vein was punctured with gentle pressure and twisting motion with a needle at the sinus level^52^, blood was collected in 1.5 ml Eppendorf tubes. Blood was collected from 4 mice at three different time points: GBM-baseline (15 days before GL261 injection), GBM-T1 (12 days after tumour induction) and GBM-T2 (21 days after GL261 implantation). T1 is a pre-symptomatic stage and T2 as a post-symptomatic stage of the disease^53^. Blood was also collected from three control mice.

Serum was prepared by allowing the blood to clot at room temperature for 30 min, followed by centrifugation at 2000 × *g* for 20 min at 4 °C and collecting the supernatant. Approximately 70 µl of serum were obtained from each blood sample. Plasma samples were prepared as following: blood was collected in a refrigerated 1.5 ml tube containing 5 µl EDTA solution (0.5 M, pH 8), centrifuged at 2000 × *g* for 20 min at 4 °C; the supernatant was then collected. Approximately 100 µl of plasma were obtained from each blood sample. The serum and plasma samples were then stored at − 80 °C.

### Sample pre-treatment

Commercial Normal Mouse Serum (Thermo Fischer Scientific) was used to compare sEV purification procedures and for all optimization steps. Aliquots of Normal Mouse Serum (100 µl and 50 µl) were prepared and stored at − 20 °C until use. All other serum and plasma samples were collected as described above. All serum and plasma samples used in this study were pre-treated by centrifugation at 4000 × *g* for 30 min at 4 °C and filtered through 0.22 µm spin filters (centrifugation at 16,000 × *g* for 1 min at 4 °C) to remove cell debris and larger EVs, respectively.

### sEV isolation by precipitation

sEV purification by precipitation was performed using the Total Exosome Isolation reagent. 20 µl of the reagent were added to 100 µl of Normal Mouse Serum and mixed by vortexing until a homogeneous solution was obtained. The solution was left to incubate at 4 °C for 30 min, and then centrifuged at 10,000 × *g* for 10 min at 4 °C. The supernatant was discarded and the resulting pellet, which contains the vesicles, was suspended in 100 µl phosphate-buffered saline (PBS) for downstream analysis. PBS was previously filtered through 0.22 µm membrane.

### sEV purification by size exclusion chromatography

SEC columns were equilibrated with PBS (previously filtered with a 0.22 µm membrane and sonicated to remove air bubbles). 100 µl of pre-treated serum were loaded on to the SEC columns (50 µl serum/plasma mixed with 50 µl of PBS for the scaled down procedure). Vesicles were eluted using PBS buffer. Fractions of 200 µl were collected. The column void volume corresponds to the first eluted millilitre (fractions 1 to 5), followed by sEV's and then proteins.

Preliminary LC–MS/MS measurements were used to establish a suitable balance between a sufficient amount of protein for LC–MS/MS analysis and sEV purity as described in detail in the Supplementary Information (Supplementary Methods S2, Supplementary Fig. S1, Supplementary Table S1).

### Ultrafiltration, buffer exchange and on-filter lysis

sEV-suspensions obtained by both precipitation (100 µl) and SEC (600 µl) were charged on 3 kDa MWCO spin filters. The samples were concentrated and rinsed two times with PBS (14,000 × *g* at 4 °C). Buffer exchange was than performed by three additions (2× 200 µl then 1× 100 µl) of an MS-compatible lysis buffer (LB) containing 1% sodium dodecyl sulfate (SDS), 5 mM ethylenediaminetetraacetic acid (EDTA), 5 mM ethylene glycol-bis(β-amino-ethyl ether)-N,N,N′,N′-tetraacetic acid (EGTA), 10 mM 4-(2-hydroxy-ethyl)-1-piperazineethanesulfonic acid (HEPES) pH 8.5 and protease inhibitor (cOmplete, Mini, EDTA-free inhibitor Mixture). Note that addition of the lysis buffer and subsequent centrifugation steps were performed at 15 °C to avoid SDS precipitation. The buffer-exchanged samples were then concentrated to a final volume of 50 µl and the sEV’s lysed on the filter by sonication using a Bioruptor Pico (Diagenode, Seraing, Belgium; 10 cycles of 30 s ON and 30 s OFF, 4 °C). The sEV-lysate was then recovered and stored at − 20 °C in 0.5 ml LoBind tubes (Eppendorf) for next day LC–MS/MS analysis.

### Proteomics sample preparation

Protein digestion was performed using a modified single-pot, solid-phase-enhanced (SP3) sample preparation protocol^54–56^. A detailed description of the protocol is available in the Supplementary Information (Supplementary Methods S3). Briefly, the sEV lysate was thawed and mixed with 1:1 trifluoroethanol together with paramagnetic beads. The lysate-bead mixture was then sonicated using the Bioruptor Pico and proteins denatured by incubation at 95 °C for 5 min. The protein mixture was then reduced, alkylated and washed, and the protein concentration determined using a modified micro BCA assay^54^. Proteolytic digestion was performed overnight at 37 °C using a Trypsin/Lys-C mixture (Promega) and a protease:protein ratio of 1:25. Further improvements to the digestion were investigated by adding a second digestion step using (i) Try/Lys-C in a protease:protein ratio of 1:75 in 60% acetonitrile, and (ii) PNGase F in a glycosidase:protein ratio of 1:20. An overview of the different digestion conditions is provided in the Supplementary Information (Supplementary Table S5). Finally, the proteolytic peptides were desalted, purified and stored at − 20 °C.

### nLC-MS/MS analysis

LC–MS/MS experiments were performed using an Easy-nLC 1000 coupled to an Orbitrap Fusion mass spectrometer (both Thermo Fisher Scientific, Bremen, Germany). A detailed description of the nLC-MS/MS methods is available in the Supplementary Information (Supplementary Methods S4). Briefly, the peptide digest was diluted 1:1 with 10% formic acid prior to injection. The peptides were separated using an EASY-Spray analytical column (ES803: 75 µm × 50 cm, C18, 2 µm, 100 Å; Thermo Scientific) using a flow rate of 300 nl/min. A 145-min LC gradient (1 μg of peptide digest injected) was used for the comparison of PPT-EV and SEC-EV purification methods and a 75-min gradient (0.5 μg of peptide digest injected) was used for all the SEC-EV proteolysis optimizations and the SEC-EV GBM longitudinal study. The Orbitrap Fusion was operated in data dependent Top Speed mode, with a 3 s cycle-time. MS1 scans were performed in the Orbitrap (375 to 1500 *m/z* at 120 K resolution), ions with charge states from 2 + to 7 + and intensity greater than 5e3 were selected for HCD fragmentation and MS2 scans were acquired in the ion trap using a 1.6 *m/z* isolation window.

### Protein identification

Raw data files were processed using Proteome Discoverer 2.1 (Thermo Scientific). The LC–MS/MS data were searched against the UniProt *Mus Musculus* protein database (January 2018, 16,945 entries), supplemented with a home-made common contaminant database (250 sequences). MS/MS spectra were searched with SequestHT search engine^57^ using the following settings: 10 ppm precursor mass tolerance and 0.6 Da fragment mass tolerance; up to 2 missed cleavages; minimum peptide length 7 amino acids; methionine oxidation (+ 15.995 Da) and acetyl (+ 42.01 Da, N termini) as dynamic modifications; cysteine carbamidomethylation (+ 57.021 Da) as fixed modification. The search engine results were then filtered for 1% false discovery rate (FDR) using the Percolator algorithm^58^ and filtered for a minimum peptide Xcorr score of 1.8. At least one unique peptide was required for definitive protein identification.

MaxQuant software (version 1.6.3.4)^59,60^ was used for the longitudinal label-free analysis of serum sEV’s from GBM-bearing mice. MS/MS spectra were searched using the Andromeda search engine using the following settings: trypsin digestion; up to 2 missed cleavages; cysteine carbamidomethylation as fixed modification; methionine oxidation and protein N-term acetylation as variable modifications; mass tolerance 20 ppm and 4.5 ppm for the first and main search respectively. Match between runs was enabled using a 0.7-min retention-time alignment. Peptides were filtered to a minimum length of 7 amino acids and 1% FDR. Raw intensities extracted from MaxQuant were used to compare sEV protein levels.

### Data analysis

Lists of protein groups identified in the PPT-sEV and SEC-sEV datasets were compared using Venn diagrams (http://bioinformatics.psb.ugent.be/webtools/Venn/). The identified protein groups were also compared with the EV markers present in the ExoCarta Top100 database^32,61^, the EVpedia Mouse database^33^, and the 2018 guidelines of the International Society of Extracellular Vesicles^3^ to verify the effectiveness of the sEV purification methods. This comparison was performed by string search of the protein names. The results are reported as the number of proteins identified that are in common with the database (ExoCarta Top100), the percentage coverage (EVpedia), and the gene names of the common proteins (MISEV2018).

Gene ontology (GO) analysis was performed using Database for Annotation, Visualization and Integrated Discovery (DAVID) v6.8 with the whole *mus musculus* genome as statistical background, and which involves a Fisher's exact test followed by an FDR multiple testing correction^62^. All proteomics data was analyzed using Perseus 1.5 software^63^, Microsoft Excel, GraphPad Prism v5 for Windows (GraphPad Software, La Jolla California USA, www.graphpad.com) and Matlab (ver. R2016a, The MathWorks Inc: Natick, MA, USA, 2010).

Note that for all analyses missing values were not replaced. Common contaminant proteins were eliminated prior to data normalization. Raw protein intensities were log2 transformed and median normalized. Principal component analysis (PCA) was performed using Perseus to investigate the principal source of variances between the datasets, and the Pearson correlation calculated to assess reproducibility. Protein groups identified in the longitudinal study of the GBM mouse model were first filtered by eliminating serum albumin and immunoglobulins, data were then log2 transformed and normalized by median subtraction, a linear mixed effect (LME) model was then applied for the three different time points. The LME model is described in detail in the Supplementary Methods S5. Error bars in graphical data represent mean ± standard deviation. All experiments were performed in biological triplicate. Statistical significance was determined by mean comparisons, using a Student’s t-test (α = 0.05) when the data (or residual) was normally distributed, verified with the Shapiro–Wilk test, or a Mann–Whitney test (α = 0.05) when the data did not exhibit a normal distribution.

### Characterization of sEV dimensions and morphology

Transmission Electron Microscopy (TEM) and Dynamic Light Scattering (DLS) measurements were performed on the sEV's isolated by SEC and PPT. sEV's were purified by SEC/PPT in duplicate from different 100 µl aliquots of serum and then concentrated on 3 kDa spin filters. The sEV samples were then washed 3 times (50% PBS/H_2_O) on the filter to remove any contaminants and decrease salt concentrations and stored at − 20 °C overnight.

#### TEM

Samples were prepared using a two-step protocol for negative staining^64^. The sEV suspensions were adsorbed for 30 min onto carbon-coated 300 mesh copper grids (Electron Microscope Science, Hatfield, PA, USA), washed three times with pure water, and then stained for 30 s with an uranium free staining solution^65^. The grids were then paper-drained and directly analysed with a Libra 120 Plus transmission electron microscope, operating at 120 kV and equipped with an in-column omega filter and 16-bit CCD camera (Zeiss, Oberkichen, Germany). Samples were analysed with ImageJ software (NIH).

#### DLS

50 µl of sEV suspension were diluted to 1 ml with PBS buffer, mixed well and loaded into a polystyrene cuvette. Each sample was analysed in triplicate at 25 °C using a Zetasizer Nano S (ZEN 1600, Malvern Instruments Ltd, UK) and a scattering angle detection of 173°. The instrument was calibrated using polymer latex spheres (Malvern).

## Supplementary information

Supplementary Information 1.

Supplementary Information 2.

Supplementary Information 3.

Supplementary Information 4.

Supplementary Information 5.

## Data Availability

We have submitted all relevant experimental data to the EV-TRACK knowledgebase (EV-TRACK ID: EV190099)^66^. The mass spectrometry proteomics data have been deposited to the ProteomeXchange Consortium (http://proteomecentral.proteomexchange.org) via the PRIDE partner repository^67^ with the dataset identifier PXD016473. All identified proteins and deregulated proteins are available in the Supplementary Datasets SD1–SD4. Any further questions or requests should be made to the corresponding author.
