# Supplementary material for: Proteomics analysis of serum small extracellular vesicles for the longitudinal study of a glioblastoma multiforme mouse model
Source: Sci Rep. 2020 Nov 24;10:20498. doi: 10.1038/s41598-020-77535-8 (PMC7686310; doi:10.1038/s41598-020-77535-8)
Supplement: Supplementary file 1 — Supplementary Information 1. [file 41598_2020_77535_MOESM1_ESM.pdf]

Supplementary information

Proteomics analysis of serum small extracellular vesicles for the longitudinal study of a glioblastoma multiforme mouse model

Federica Anastasi[1,2], Francesco Greco[2,3], Marialaura Dilillo[2], Eleonora Vannini[4,5], Valentina Cappello[6], Laura Baroncelli[4,7], Mario Costa[4], Mauro Gemmi[6], Matteo Caleo[4,8], Liam A. McDonnell[2, *]

[1] NEST laboratories, Scuola Normale Superiore, 56127 Pisa, Italy
[2] Fondazione Pisana per la Scienza ONLUS, 56107 San Giuliano Terme (PI), Italy
[3] Institute of Life Sciences, Sant'Anna School of Advanced Studies, 56127 Pisa, Italy
[4] CNR, Neuroscience Institute, 56124 Pisa, Italy
[5] Fondazione Umberto Veronesi, 20122 Milano, Italy
[6] Istituto Italiano di Tecnologia, Center for Nanotechnology Innovation @NEST, 56127 Pisa, Italy
[7] IRCCS Fondazione Stella Maris, 56018 Calambrone (PI), Italy
[8] Department of Biomedical Sciences, University of Padua, 335122 Padua, Italy

* Corresponding author: liam@fpscience.it

Supplementary Information summary

Supplementary Methods 3
Methods S1. Mice Tumor induction............ 3
Methods S2. Size exclusion chromatography sEV's fraction establishment 3
Methods S3. Proteomics sample preparation 4
Methods S4. nLC-MS/MS analysis............ 4
Methods S5. Data analysis - Linear mixed effect model (LME) 6
Supplementary Results 6
Results S1. Comparison of serum and plasma sEV............ 7
References............ 7
Supplementary Figures............ 8
Figure S1. Size exclusion chromatography elution profile analysis. 8
Figure S2. Comparison of sEV proteins from 50 µl serum and 50 µl plasma. 9
Supplementary Tables............ 9
Table S1. LC-MS/MS proteomics analysis of the SEC elution fraction pools. 9
Table S2. EV proteins identified from PPT and SEC preparations.. 10
Table S3. Gene name of identified proteins in common with ExoCarta Top 100 database..... 10
Table S4. Gene Ontology Enrichment Analysis............ 11
Table S5. Digestion optimization conditions 11
Table S6. Proteomics data of the 25 significantly deregulated proteins 12

Supplementary Methods

Methods S1. Mice Tumor induction

The murine glioma GL261 cell line were grown as specified in Vannini et al., 2014 [1]. C57BL/6 mice were anesthetized with avertin (intraperitoneal injection of 2,2,2-tribromoethanol solution; 250 mg/kg body weight). The tumour was induced by stereotaxically guided injection of 40,000 GL261 cells (20,000 cells/1 µl Tris HCl solution) into the primary motor cortex (1.75 mm lateral and 0.5 mm anterior to bregma). Body temperature was constantly monitored with a rectal probe and maintained at 37.0°C with a thermostatic electric blanket during the surgery. An oxygen mask was placed in front of the animal's mouth to aid respiration. The GL261 cell solution was slowly delivered at a depth of 0.8–0.9 mm from the pial surface. To prevent dehydration, a subcutaneous injection of saline (0.9% NaCl, 1 ml) was delivered at the end of the procedure.

Methods S2. Size exclusion chromatography sEV's fraction establishment

A 100 µl aliquot of normal mouse serum was loaded onto the SEC column and twenty-five 200 µl fractions collected. These fractions were pooled to ensure there was sufficient protein content for LC-MS/MS analysis. Four pools were selected and analyzed on the basis of the SEC column elution profile suggested by the manufacturer (https://www.schaefer-tec.it/sites/default/files/qEVsingle_Technical_Note.pdf). These were EV-1 (fractions 6 to 8), EV-2 (fractions 9 and 10), PROT-1 (fractions 11-17), and PROT-2 (fractions 18-25). The lists of proteins identified from each pooled-sample were then compared with the proteins contained in the ExoCarta Top100 database [2]. Sample EV-1 contained the highest number of EV-proteins, which was more than double that identified from any other SEC sample and also contained the most unique protein identifications (Figure S1). The total protein amount extracted from SEC sample EV-2 indicated serum protein contamination, 3.5 µg in comparison to 1.8 µg for EV-1 (Table S1). Serum proteins also dominated the late SEC eluate samples Prot-1 and Prot-2 (Figure S1). In view of the higher purity of the sEV's all subsequent SEC experiments were performed using the EV-1 protocol.

Methods S3. Proteomics sample preparation

The sEV lysate was obtained by on-filter buffer exchange and on-filter sEVs lysis. Protein digestion was performed using a modified SP3 protocol[3],[4],[5] that was further adapted in this work owing to the higher sample volume (sEV lysate collected from 3KDa filter was approximately 50 µl). The protein extract solution was mixed with trifluoroethanol (TFE) in a 1:1 ratio and 2 µl of carboxylate coated paramagnetic beads (100 mg/ml solution of 50% Speedbeads A (GE65152105050250, Sigma) and 50% Speedbeads B (GE65152105050250, Sigma) in 0.5 ml Protein LoBind tubes (Eppendorf AG, Hamburg, Germany). To promote a more complete lysis the sample was sonicated for 10 min (30 sec on/off cycles using a Bioruptor Pico (Diagenode, Belgium) at 4°C. Proteins were then denatured by incubation at 95°C for 5 minutes. Reduction and alkylation were performed by adding 200 mM DTT (1 µl each 20µl LB-TFE solution) for 30 min at 45°C, followed by 400 mM iodoacetamide (same amount of DTT) for 30 min at RT in the dark and then quenched with 200 mM DTT (same amount of DDT and IAA). Proteins were then immobilized on the beads by the addition of 50% acetonitrile (ACN). The beads-bound-proteins were rinsed twice with 70% ethanol and once with 100% ACN. The protein-bound-beads were dried and proteins eluted by the addition of 10 µl 50 mM Hepes pH 8 and sonicated for 5 min at RT (30 sec on/off cycles). The total protein content in each sample was then quantified using 1 µl aliquots and a modified microBCA assay[3]. Enzymatic digestion was performed overnight (18h) at 37°C in 12 µl by adding Trypsin/Lys-C mixture (1:25 enzyme/protein). Digestion was optimized in this work by testing two different digestion conditions (Table-S3). Peptides were then purified by the addition of ACN up to 95% and rinsed with 100% ACN. Beads were than dried and purified peptides were eluted from the beads with a 2% DMSO aqueous solution. Samples were then stored at -20°C for downstream LC-MS/MS analysis.

Methods S4. nLC-MS/MS analysis

Purified peptide digests were diluted 1:1 with 10% formic acid prior to their injection on an Easy-nLC1000 (Thermo Scientific) coupled to an Orbitrap Fusion (Thermo Scientific). Peptides were separated in reverse phase mode online using an EASY-Spray PepMap^{TM} analytical column (ES803: 75 µm x 50 cm, C18, 2 µm, 100 Å; Thermo Scientific) at a flow rate of 300 nl/min equipped with an Acclaim PepMap^{TM} trap column (75 µm x 2 cm, C18, 3 µm, 100 Å; Thermo

Scientific). A peptide mixture from bovine serum albumin (Sigma-Aldrich; St. Louis, MO) was used as a quality control to assess carry-over and to test the overall chromatographic performance. A QC experiment was performed after every sEV analysis. Two different LC gradients were utilized to separate the tryptic peptides: a 145 min gradient (for PPT/SEC comparison and 100-to-50 µl scale down study) and a 75 min gradient (SEC-EV optimization and SEC-EV GBM longitudinal study).

**Gradient**	Peptide load	0 min	1 min	53 min	60 min	66 min	75 min
**75 min**	0,5 µg	6% B	6% B	23% B	33% B	90% B	90%
**Gradient**	Peptide load	0 min	1 min	105 min	120 min	130 min	145 min
**145 min**	1 µg	5% B	5% B	22% B	32% B	90% B	90%

Buffer A consisted of LC-MS grade water with 0.1% formic acid and Buffer B of LC-MS grade acetonitrile with 0.1% formic acid. Peptides were loaded at 800 bar and then separated using one of the above non-linear gradients.

The Orbitrap Fusion Tribrid mass spectrometer operated in positive ion mode. The nESI voltage was 2100-2300 V and the ion transfer tube temperature was 275 °C. Spectra were acquired in Top Speed mode using a 3 seconds cycle time. The MS method consisted of a full mass spectrum in the Orbitrap (scan range: 375 to 1500 *m/z*, 120 K resolution, AGC target of 4e5, max. injection time 100/50 ms for the 75/145 min gradient respectively). Monoisotopic precursor selection and a dynamic exclusion of 60s were adopted. Ions with charge states from 2+ to 7+ and intensity greater than 5e3 were selected for HCD fragmentation at 30% NCE using an isolation window of 1.6 *m/z*. MS/MS acquisition were performed in the linear ion trap with a rapid scan rate, AGC target of 1e4 (2e3 for 145 min gradient) and 35 ms maximum injection time (300 ms for 145 min gradient).

Methods S5. Data analysis - Linear mixed effect model (LME)

For the longitudinal study, identified protein groups were filtered by eliminating serum albumin, and immunoglobulins, whose intensities may depend on the serum preparation and SEC elution. The data were then log2 transformed and normalized by median subtraction. An ANOVA test could not be used for the comparison between the three different time points, because the samples are not independent (longitudinal analyses explicitly concern the time dependence of the signals). A linear mixed effect (LME) model was applied and Matlab was used for model fitting. The time effect was set as a fixed categorical variable with three levels (GBM-baseline, GBM-T1 and GBM-T2). A random intercept for each mouse was added to the model, to account for inter-individual variability of each protein at baseline. For this reason, the LME model is often utilized for longitudinal studies [6,7]. The LME model was fitted for each protein group if a sufficient number of three valid values were present for each time point and if the residuals and the random effect were normally distributed (Shapiro-Wilk test, α=0.05). The LME model equation is:

$$y_{k,j}=(\beta_0+\nu_{0,k})+\beta_{1,j}\tau_j+\varepsilon_{k,j}$$

in which the intensity of the protein ($y_{k,j}$) is predicted by the fixed effect of the time point τ_j and the random effect $\nu_{0,k}$ of the mouse k. The model was tested for significant effects and the p-values were corrected to an FDR of 0.05 using the Benjamini-Hochberg method. Significant time point effects were plotted with their confidence interval at 95%. The sEV proteome of three control and three GBM-T2 mice were also compared using a two-sided Student's t-test and the p-values were also corrected to an FDR of 0.05 using the Benjamini-Hochberg method. All proteins with a corrected p-value <0.05 were considered significant.

Supplementary Results

Results S1. Comparison of serum and plasma SEC-EV proteins

During the preparation of serum the fibrin clot incorporates a large number of blood proteins [8], and extracellular vesicles may become trapped within this network [9]. Differing amounts of fibrin during clotting could thus affect the number of EVs that can be isolated from serum. To assess if this source of variability had an effect on the analysis of EV proteins the SEC-EV isolation procedure was applied to 50 µl serum and 50 µl plasma samples from the same mice (n=3), the proteins were then extracted from the sEV's and digested using the two-step digestion procedure. The number of identified proteins was much more variable for the plasma derived sEV, with 213±4 protein groups identified from serum-sEV and 206±88 from plasma-sEV, Figure S2A. Protein intensities were also more variable for the plasma-sEV. Figure S2B shows the result of principal component analysis (PCA) performed on the log2 transformed, median normalized data. The PCA score plot shows that the two sample types are separated by PC1, and that the serum sEV samples from different animals were clustered together more closely than the plasma sEV samples. The loading plot shows that three proteins were primarily responsible for the separation of plasma sEV's from serum sEV's, Figure S2C, and were all part of the fibrinogen family (Fga, Fgb, Fgg).

References

[1] Vannini, E. *et al.* The bacterial protein toxin, cytotoxic necrotizing factor 1 (CNF1) provides long-term survival in a murine glioma model. *BMC Cancer* 14, 449 (2014).

[2] Keerthikumar, S. *et al.* ExoCarta: A Web-Based Compendium of Exosomal Cargo. *J. Mol. Biol.* 428, 688–692 (2016).

[3] De Graaf, E. L., Pellegrini, D. & McDonnell, L. A. Set of Novel Automated Quantitative Microproteomics Protocols for Small Sample Amounts and Its Application to Kidney Tissue Substructures. *J. Proteome Res.* 15, 4722–4730 (2016).

[4] Hughes, C. S. *et al.* Ultrasensitive proteome analysis using paramagnetic bead technology. *Mol. Syst. Biol.* 10, 757 (2014).

[5] Pellegrini, D. *et al.* Quantitative Microproteomics Based Characterization of the Central and Peripheral Nervous System of a Mouse Model of Krabbe Disease. *Mol. Cell. Proteomics* 18, 1227–1241 (2019).

[6] Liu, C. W. *et al.* Temporal profiles of plasma proteome during childhood development. *J. Proteomics* 152, 321–328 (2017).

[7] Carlsson, A. C. *et al.* Use of proteomics to investigate kidney function decline over 5 years. *Clin. J. Am. Soc. Nephrol.* 12, 1226–1235 (2017).

[8] Talens, S., Leebeek, F. W. G., Demmers, J. A. A. & Rijken, D. C. Identification of Fibrin Clot-Bound Plasma Proteins. *PLoS One* 7, e41966 (2012).

[9] Stachowicz, A. *et al.* Optimization of quantitative proteomic analysis of clots generated from plasma of patients with venous thromboembolism. *Clin. Proteomics* 14, 38 (2017).

Supplementary Figures

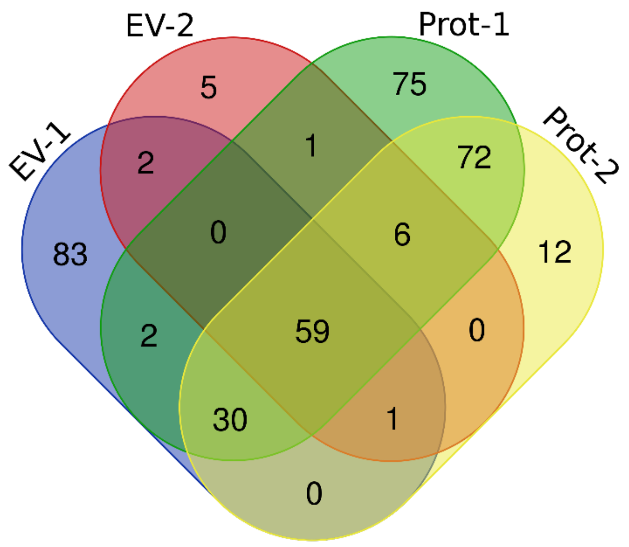

Fractions pool	# IDs	ExoCarta Top 100
EV-1	177	22
EV-2	74	5
Prot-1	245	9
Prot-2	180	6

Figure S1. Size exclusion chromatography elution profile analysis.
LC-MS/MS analysis was performed on the four SEC elution fractions: EV-1, EV-2, PROT-1, PROT-2. (A) Venn diagram showing overlap between the protein groups. (B) Number of protein groups for each SEC elution fraction and coverage of ExoCarta mouse Top 100 database.

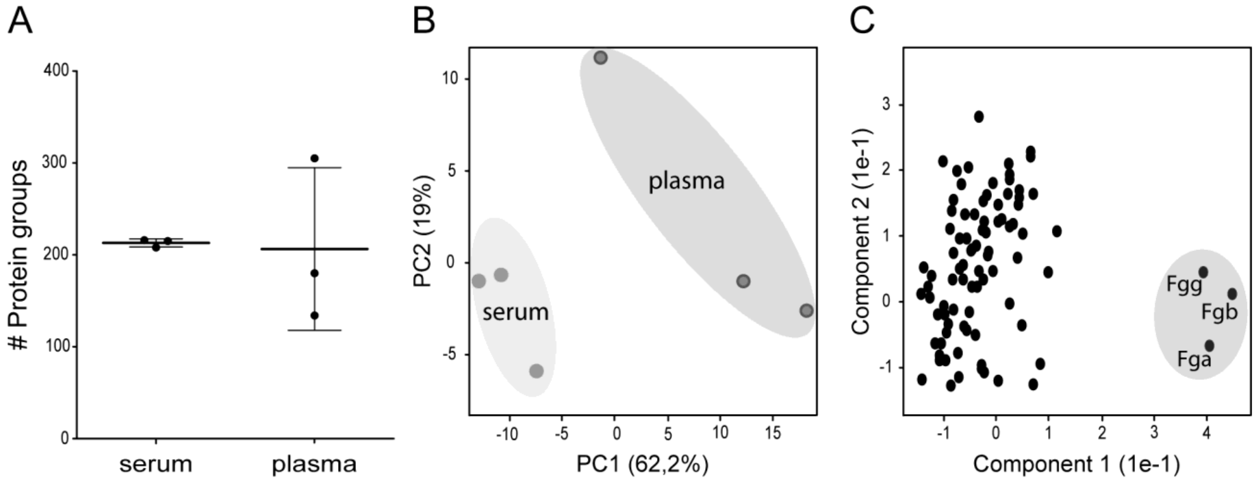

Figure S2. Comparison of sEV proteins from 50 µl serum and 50 µl plasma.
(A) Number of identified protein groups. (B) PCA score plot, performed on the filtered and normalized protein intensities, clearly separates the serum and plasma datasets. (C) PCA loading plot demonstrates that the separation of the plasma and serum derived sEV's was due to fibrinogen proteins (Fga, Fgb and Fgg).

Supplementary Tables

Table S1. LC-MS/MS proteomics analysis of the SEC elution fraction pools.

	**EV-1**	**EV-2**	**Prot-1**	**Prot-2**
SEC Fractions	6-8	9-10	11-17	17-25
Protein amount (µg)	3.5	8	2232	1764
Protein groups (# IDs)	177	74	245	180
Peptide groups #	827	214	2136	1241
PSMs	1383	15849	27327	24448
0 Missed cleavages	57.98%	80.40%	88.43 %	97.12 %
1 Missed cleavages	34.85 %	19.18%	10.82%	2.78 %
2 Missed cleavages	7.15 %	0.4 %	0.73 %	0.09 %

Table S2. EV proteins identified from PPT and SEC preparations. The table reports the presence of known EV markers according to the MISEV 2018, ExoCarta Top 100 and EVpedia databases. Only proteins identified in all replicates were used for this comparison.

Identified proteins expected to be present in EVs (MISEV 2018)		
**Category**	PPT	SEC
**Transmembrane protein associated to plasma membrane and/or endosomes**	-<br>MHC class I (H2-Q10)<br>-	Tetraspanins (Cd82, Cd151)<br>MHC class I (H2-K1, H2-D1)<br>Integrins (b1,b2,b3,a6); Adam10
**Cytosolic proteins recovered in EVs**	Anxa2, Hspa8, Gapdh	Ehd-1, Ehd-4, Hspa8, Actc1, Tuba1a, Tubb4b, Gapdh, Sdcbp
**Adhesion and extracellular matrix proteins**	Fibronectin, Collagen (Col12a1), Ahsg, Cd5l Galectin-3-binding protein	Tgfb1, Fibronectin, Mfge8, Lgals3bp, Cd5l, Ahsg, Collagen (Col1a1, Col1a2)
**Exosomes database coverage**		
**ExoCarta Top 100**	14	25
**EVPedia**	91.1%	93.7 %

Table S3. Gene name of identified proteins also present in ExoCarta Top 100 database. Only proteins identified in all replicates are reported.

ExoCarta Top 100	
**Precipitation**	Thbs1; Anxa2; Hsp90aa1; Hspa8; Msn; Ldha; Flna; Hspa5; Alb; Tfrc; Myh9; A2m; Lgals3bp; Gapdh
**Size exclusion chromatography**	Vcp; Slc3a2; Aldoa; Stom; Ldha; Flna; Alb; Tfrc; A2m; Bsg; Thbs1; Sdcbp; Itgb1; Itga6; Hspa8; Slc16a1; Atp1a1; Tpi1; Prdx2; Rap1b; Myh9; Ywhaz; Gapdh; Acly; Ezr

Table S4. Gene Ontology Enrichment Analysis of identified proteins of PPT and SEC datasets, using DAVID database v.6.8.

	PRECIPITATION			SIZE EXCLUSION CHROMATOGRAPHY		
	GO term	COUNT	FDR	GO term	COUNT	FDR
**Cellular component**	Extracellular region	176	3,3E-113	Extracellular exosome	255	5,8E-110
	Blood microparticle	78	4,3E-108	Blood microparticle	76	1,27E-87
	Extracellular space	163	1,9E-107	Extracellular region	181	2,2E-75
	Extracellular exosome	183	9,4E-90	Extracellular space	160	1,3E-66
	Extracellular matrix	45	1,3E-29	Extracellular matrix	53	2,5E-29
	High-density lipoprotein particle	13	1,3E-14	Focal adhesion	48	8,1E-19
	Immunoglobulin complex	14	3,6E-14	Cell surface	57	1,5E-16
	Cell surface	7	6,1E-10	Cell-cell adherens junct.	39	8,3E-15
	External side of plasma membrane	37	3,1E-9	Extracellular vesicle	18	1,6E-13
	Fibrinogen complex	26	6,0E-8	Myelin sheath	3	1,6E-13

Table S5. Digestion optimization conditions

	Conditions	Before optimization	II step - PNGase	II step – ACN/Try/Lys-C
**I step**	Time (hours)	18	16	16
	Enzyme	Try/Lys- C	Try/Lys- C	Try/Lys- C
	Enzyme:Protein ratio	1:25	1:25	1:25
	Digestion volume (µl)	12	12	12
	Digestion solution	50 mM HEPES	50 mM HEPES	50 mM HEPES
**II step**	Time (hours)	-	2	2
	Enzyme		PNGase	Try/Lys-C
	Enzyme: Protein ratio		1:20	1:75
	Digestion volume (µl)		14	30
	Digestion solution		50 mM HEPES	60% ACN- 40% Hepes 50 mM

Table S6. Proteomics data of the 25 significantly deregulated proteins identified in the longitudinal analysis of the GBM mouse model.

Gene name	Protein name	Uniprot Accession #	MS/MS count	Unique Peptides	Sequence Cov. %	MW (kDa)	Score
Thbs1	Thrombospondin-1	P35441	680	73	55.6	129.65	323.31
Apoc4	Apolipoprotein C-IV	Q61268	69	5	25	14.288	117.78
C4bpa	C4b-binding protein	P08607	180	13	36.2	51.523	279.94
C1ra	Complement C1r-A subcomponent Complement C1q	Q8CG16	277	33	62.5	80.072	323.31
C1qa	Complement C1q subcomponent subunit A	P98086	70	8	34.3	25.974	155.2
C1sa	Complement C1s-A subcomponent Transferrin receptor	Q8CG14	119	19	58	76.857	323.31
Tfrc	Transferrin receptor protein 1	Q62351	80	17	28.3	85.73	117.29
Lrp1	Prolow-density lipoprotein receptor-related protein 1	Q91ZX7	83	30	10.5	504.74	114.93
Itih1	Inter-alpha-trypsin inhibitor heavy chain H1	Q61702	77	17	25.2	101.07	148.54
Apoc3	Apolipoprotein C-III	P33622	20	2	38.4	10.982	155.62
Angpt1	Angiopoietin-1	O08538	62	18	39	57.518	118.26
Uba52	Ubiquitin-60S ribosomal protein L40	P62984	21	3	21.1	14.728	19.092
Pm20d	N-fatty-acyl-amino acid synthase/hydrolase PM20D1	Q8C165	27	7	19.3	55.662	54.11
Itga2b	Integrin alpha-IIb	Q9QUM0	29	9	14	112.68	23.023
Myh9	Myosin-9	Q8VDD5	127	42	28.6	226.37	309.54
Cpn1	Carboxypeptidase N catalytic chain	Q9JJN5	13	6	15.5	51.845	28.667
Vtn	Vitronectin	P29788	49	8	27.6	54.848	98.666
Itih3	Inter-alpha-trypsin inhibitor heavy chain H3	Q61704	31	10	17.3	99.357	43.623
Glul	Glutamine synthetase	P15105	8	3	14.5	42.119	6.7318
Tln1	Talin-1	P26039	12	9	6.1	269.82	15.191
Fbln1	Fibulin-1	Q08879	13	9	19.6	78.032	15.446
Vcan	Versican core protein	Q62059	5	3	1.3	366.78	4.5883
Flna	Filamin-A	Q8BTM8	10	8	5.8	281.22	13.638
Lamb1	Laminin subunit beta-1	P02469	23	12	10	197.09	25.175
Pcyox1	Prenylcysteine oxidase	Q9CQF9	5	6	21	56.494	15.824
